# *It’s a small bit of advice, but actually on the day, made such a difference…*: perceptions of quality in abortion care in England and Wales

**DOI:** 10.1186/s12978-021-01270-0

**Published:** 2021-11-07

**Authors:** Katherine C. Whitehouse, Rebecca Blaylock, Shelly Makleff, Patricia A. Lohr

**Affiliations:** 1 Centre for Reproductive Research & Communication, BPAS, 30-31 Furnival Street, London, EC4A 1JQ UK; 2 School of Public Health and Preventive Medicine, Monash University, 553 St Kilda Road, Melbourne, VIC 3004 Australia

**Keywords:** Abortion, Termination of pregnancy, Quality of care, Qualitative research, Health services research, Quality improvement

## Abstract

**Background:**

Quality of care (QOC) is increasingly identified as an important contributor to healthcare outcomes, however little agreement exists on what constitutes quality in abortion care or the recommended indicators from the service-user perspective. Our study aimed to explore perceptions and experiences of abortion QOC in England and Wales.

**Methods:**

We performed in-depth interviews (via phone or in-person) with participants who had an abortion at a nationwide independent sector provider in the previous 6 months. We explored their experiences of the abortion service at each point in the care pathway, their perspectives on what contributed to and detracted from the experience meeting their definitions of quality, and their reflections on different aspects of QOC. We used content analysis to generate themes.

**Results:**

From December 2018 to July 2019, we conducted 24 interviews. Ten participants had a surgical and 14 had a medical abortion. Seventeen (71%) were treated in the first 12 weeks of pregnancy and 7 (29%) beyond that, with an average gestational age of 10 weeks + 5 days (range 5–23 + 6). We identified 4 major themes that contributed to participant’s perception of high quality care: (1) interpersonal interactions with staff or other patients, (2) being informed and prepared, (3) participation and choices in care and (4) accessibility. Nearly all participants identified interpersonal interactions with staff as an important contributor to quality with positive interactions often cited as the best part of their abortion experience and negative interactions as the worst. For information and preparation, participant described not only the importance of being well prepared, but how incongruencies between information and the actual experience detracted from quality. Participants said that making choices about their care, for example, method of abortion, was a positive contributor. Finally, participants identified access to care, specifically in relation to waiting times and travel, as an important aspect of QOC.

**Conclusions:**

Participants situated quality in abortion care in 4 domains: interpersonal aspects of care, information and preparation, choices, and accessibility. Indicators identified can be used to develop standard metrics to ensure care meets service-user needs.

## Plain English summary

Quality of care is an important aspect of delivering healthcare in a patient-centred manner. There is a lack of agreement about what consititutes quality care for an abortion service. In our study, we interviewed 24 participants who had an abortion in the last 6 months to assess their perceptions of quality of care. Based on our analysis, we identified 4 themes that were important contributors to quality: (1) interactions with clinic staff or other patients, (2) information and preparation for the abortion, (3) making choices about care, and (4) access to abortion care. Almost all of our study participants said that positive interactions with clinic staff contributed to a quality experience. Participants wanted to be well-prepared for their abortion. They reported a negative experience when the information they were given did not match their experience. Participants felt that active participation in choices around their abortion experience improved quality. Finally, ease of access to abortion care made for a more positive experience. Abortion providers and researchers should consider using the results of our study to ensure their services meet patients’ needs.

## Introduction

Quality of care (QOC) is a fundamental aspect of healthcare provision, and its measurement informs efforts to improve health services of different types. The World Health Organization (WHO) defines QOC as “the extent to which healthcare services provided to individuals and patient populations improve desired health outcomes” [1]. To achieve this, the WHO states that services must be safe, effective, timely, efficient, equitable and person-centred. Standards and frameworks drawing on these domains of quality have been articulated for different types of sexual and reproductive health care [2–4], but little agreement exists on what constitutes quality in abortion care [5]. Researchers, health agencies, and professional societies have proposed a large number of indicators to assess the quality of abortion services. However, definitions and applications of these indicators are inconsistent across organisations and contexts and tend to emphasis clinical aspects of care rather than patient perspectives [5].

In 2017, a group of stakeholder organisations came together to launch the Abortion Service Quality (ASQ) Initiative [6]. Their aim was to develop the first ever global standard for measuring the quality of abortion services in low- and middle-income countries (LMIC). In addition to indicators of safety and technical quality, they identified a need to develop patient-centred metrics. The British Pregnancy Advisory Service (BPAS) is a member of the ASQ Initiative resource group and the largest provider of abortion care in Great Britain (GB), with a network of clinics across England and Wales. Commissioned by the National Health Service (NHS), BPAS provides abortion, contraception, and miscarriage management services to approximately 100,000 people per year. BPAS had an interest in exploring QOC in the high-income setting of Britain to complement the focus of the ASQ initiative and build on existing frameworks and standards for abortion quality in England and Wales [7–10].

Research in the UK has engaged with some facets of abortion QOC, for example, barriers to abortion services [11, 12], information needs when seeking abortion [13, 14], and preferences for particular aspects of care such as self-managed abortion [15]. However, recent data are lacking on patient perceptions of abortion QOC in Britain overall, and what specific aspects of care contribute to high-quality services. We aimed to address this gap by using in-depth interviews to explore participants' experiences and views on abortion QOC in England and Wales.

## Methods

### Setting

Independent-sector organisations perform over three-quarters of abortions in England and Wales, most of which are provided under contract to the NHS [16]. These organisations, including BPAS, may provide care from outpatient freestanding facilities or clinics within, but not integrated into, NHS hospitals or GP practices. Independent providers of abortion can only provide abortion care up to 23 weeks and 6 days of gestation, regardless of indication. However, in the NHS, abortions can be carried out over 24 weeks’ gestation for fetal anomaly or threat to maternal life-although those cases accounts for less than 2% of abortions nationwide [9]. BPAS provides 43% of abortions in England and Wales through a network of 50 clinics and five telemedicine hubs. Abortions at BPAS are provided both medically and surgically to 23 weeks and 6 days’ gestation, but only one clinic provides medical abortion over 10 weeks’ gestation.

### Data collection

We conducted one-to-one, in-depth interviews between December 2018 and July 2019 with participants from seven geographically diverse BPAS clinics in England and Wales who had had an abortion in the last six months. We recruited from clinics that performed medical and surgical abortions across a range of gestations and also those which had an overrepresentation of ethnic minority populations. Clinical staff and members of the study management team approached potential participants about the study both directly at in-person clinical visits or passively through posters displayed in the participating clinics. We attempted to purposively sample our population by approaching participants with diversity of the following characteristics: participant age, gestational age, abortion type (medical or surgical), race/ethnicity. Participants were eligible if they were over the age of 18, spoke English, and resided in Great Britain at the time of their abortion. We excluded those who had an abortion for fetal anomaly or spontaneous fetal demise/miscarriage, and those who did not speak English as we lacked the resources for translation.

We conducted interviews either in-person (in a private space at a BPAS facility), or remotely via telephone. If a participant did not attend the interview, we made one attempt to follow-up and reschedule. We obtained informed consent at the start of the interview either verbally for remote interviews or in writing for in-person interviews. Interviews took approximately 60 minutes to complete and were audio-recorded. We provided participants £20 compensation.

The interview guide explored: (1) how participants describe their experiences of QOC for their abortion, (2) how participants perceive and define abortion QOC, including which elements are most important, and (3) the relationship between abortion stigma and QOC. We concluded by asking participants to reflect on the interview and share the three most important aspects of abortion QOC from their perspective. After completing the first three interviews as a pilot, the study management team refined the instrument. Because the interview guide did not change significantly after this review, we included the pilot interviews in our overall sample. The National Research Ethics Service (ID# 251162) and BPAS Research and Ethics Committee (ID# 2018/09/KW) approved our study.

### Data analysis

Audio recordings were transcribed and imported into Dedoose for analysis [17]. We based our analysis strategy on similar work [18], drawing on QOC frameworks [1–4] to categorise the data. After we developed a list of codes, we individually coded all the transcripts. One researcher performed quality control assessments across all transcripts to ensure consistency in code application. We clustered the codes by thematic area and used thematic analysis to write code summaries [19]. Through consensus discussions, we refined the analysis and interpreted the findings across themes. We interpreted QOC in the context of participants’ descriptions of positive and negative experiences and perceptions of their abortion service, even when they didn’t mention ‘quality’ specifically.

## Results

Twenty-four participants enrolled in this study. We performed most of the interviews (n = 23) via telephone and one in-person. Participant characteristics are further detailed in Table 1. The majority of participants were white, most were employed, and in a relationship/married. Seventeen participants (71%) had an abortion in the first 12 weeks’ of pregnancy and 7 (29%) beyond that. We identified four major themes of abortion care that were meaningful for participants and influenced their perceptions of the QOC they received: (1) interpersonal interactions with clinic staff or other patients, (2) being informed and prepared, (3) participation and choices in care and (4) accessibility. In addition, we identified three minor themes including (1) confidentiality, (2) attributes of clinical facilities, and (3) staff competency. We discuss the major themes in this manuscript.

**Table 1:** Participants (N = 24) characteristics from in-depth qualitative interviews on abortion quality of care

Characteristic	n (%)
Age (mean, range)	29 (19–42) years
Race	
White	23 (95.8)
Mixed	1 (4.2)
Highest level of education achieved	
No qualification	1
GCSE	4
A-levels	5
NVQ/BTEC/College/Foundation degree	4
Undergraduate	6
Postgraduate	4
Employment status	
Employed (full- or part-time)	14 (58.3)
Unemployed	5 (20.8)
Student	7 (29.2)
Relationship status	
Single	6 (25)
In a relationship	10 (41.7)
Married/civil partnership	7 (29.2)
Separated/divorced	1 (4.2)
Obstetrical history (mean, range)	
Number of prior pregnancies (mean, range)	3.1 (1–9)
Number of prior live births (mean, range)	1.2 (0–3)
Number of prior abortions^a^ (mean, range)	1.5 (1–3)
Gestational age at time of abortion^b^ (mean, range)	10 (5 to < 24) weeks
Abortion type	
Medical	14 (58.3)
Surgical	10 (41.7)

^a^The index abortion discussed in the interview

^b^Gestational age as reported by the participant

### Interpersonal interactions

Nearly all participants described how interpersonal interactions with clinic staff influenced their care experience. They consistently reported positive interactions with staff as the best part of their abortion experience. Positive descriptors included: “friendly”, “caring”, “thoughtful”, “sympathetic”, “empathetic”, “respectful”, “non-judgmental”, and “understanding”. Following these positive interactions, participants described feeling comfortable, reassured, and supported. One participant elaborated on this:

I think she just made me feel, you know, completely like comfortable […], and like the language that she used made, just really put me at ease. I think I’d been sort of a little bit nervous when I got there; I’d seen the receptionist and she was lovely, erm, but I think that it was the second lady that I came into contact with that really sort of put my, put my mind at ease and made me feel, you know, really comfortable being there.

Even small gestures by staff had positive effects on participants. Examples included a staff member remembering a participant from a previous visit, making sure they had somewhere to park, providing refreshments during recovery, simply introducing themselves, or stroking their head during the abortion:

…she brought me…the hot chocolate and sh-she not only was friendly but she actually spoke to you and listened and she couldn’t believe that I came all the way from Edinburgh.

Conversely, participants said that negative interactions with clinic staff detracted from their abortion experience, and some ranked these interactions as the worst part of their abortion. Experiences that detracted from their abortion experience included “rude” or “unwelcoming” attitudes or “a slightly telling off kind of tone”. One participant described a staff member who “didn’t introduce herself, she didn’t smile, she didn’t say anything, it was literally just like she just wanted to do her job and get home.” Participants also described negative interactions with staff which left them feeling “criticised” or “chastised”, for example vomiting due to medication side effects: “It was a bit like she [staff member] was saying, like, ‘Oh, like […] can you try not to be sick on the floor?’ and it was like […] ‘I’m not really doing it on purpose.’”.

Participants also described how interactions with other patients in shared clinic spaces impacted their experience. Two participants said that seeing other patients in the waiting area created a sense of solidarity: “There is also something quite comforting about knowing that you’re not the only one there and that other people are going through this.” However, some participants described how shared spaces could generate a negative experience. For example, a few participants said the waiting room lacked privacy for anyone who was outwardly emotional and one participant said hearing another patient crying in the changing room was “a bit distressing”. A few participants also had concerns that they might be recognised at the clinic by someone they knew or their conversations would be overheard. Several participants described negative perceptions of support people in the waiting area: “A lot of partners are in there and they were, like, asleep and snoring, and it was, like, really weird.” Finally, a few participants described negative experiences with protestors outside clinic(s). For one participant, this was an “extra thing that nobody needs in this situation” and was among the worst parts of their abortion experience.

### Being informed and prepared

Most participants described being informed and prepared as contributors to QOC. Information provision improved their ability to participate in care decisions and anticipate how that care would be provided. Some said that being well-informed and prepared was one of the best parts of the service, and others ranked being unprepared as one of the worst parts. Participants said it was useful to receive information via several modalities, including websites, brochures, phone appointments, and verbally.

Participants positively described staff who spent sufficient time counselling them such as a nurse who “just wanted to take the time to explain things to me. I feel like I’d been given plenty of information, I wasn’t left wondering what was going to happen.” They expressed a desire to receive detailed information about “every little thing that’s going to happen”, “from the beginning to after,” and that staff should “keep informing you and telling you what’s happening along the way.” They described wanting detailed information on which sanitary towels to use after their abortion, identification of excess bleeding, access to postabortion counselling, permitted support people, and length of each part of the procedure. The desire for information extended even to seemingly minor, non-clinical advice:

[I] was given the advice […] to take a sandwich with me ‘cause […] I’d be really hungry because of not eating. And actually, I was sick after the general anaesthetic and then when I had that sandwich it actually sorted me completely out […]. It’s a small bit of advice, but actually on the day made such a difference.

Participants expressed the importance of being adequately informed about the timing of different components of the abortion service. Participants expressed frustration when they did not know how long the in-clinic wait would be, especially if a support person was with them. Some participants said they would appreciate better communication from staff throughout their visit about wait times, delays, and how long each part of the appointment would take. Some participants said they wished they had had more warning that their procedure was about to happen. One said, “I think it was a bit fast. Like, they’d call your name and then suddenly, you know, it’s the walk straight into theatre.” Two participants said that they felt rushed while taking medical abortion tablets at the clinic (as was legally required at that time before the approval of mifepristone at home), which had a negative impact on their abortion experience.

Participants described feeling anxious or unprepared if the information given did not correlate with their actual experience. As one participant said, “I get very anxious, erm, so it’s more, it was more like in my head I had that, ‘this, this and this is going to happen’ and then that one thing didn’t happen, and then it just, it throws me a bit”. In another example, a participant said she was told she could wear her own clothes during the procedure, but when she arrived at the clinic, another staff said this was not possible. Two participants described situations of feeling underprepared for the pain of medical abortion. One said staff told her she would experience a “slight niggling pain” but in reality she was in “agony”. Another said the staff compared the pain to “period-like cramps”, but as she had never experienced period pains, this was not helpful.

### Participation and choices in care

Some participants described how having choices or actively participating in decisions about their care created a positive experience. One said, “the fact that I had choices, the fact that I knew that if I wanted to change my mind I could, at any time […]. I felt kind of like, erm, I had a little bit of control over it.”

Participants described how being able to choose between a surgical and medical abortion met their needs in the following quotations:

Surgical: Because I just needed it, I didn’t want to know about it, first of all. And second of all, my, I was on my own…At home with the children, ‘cause my husband was working away…So, I had to go on my own, I had to come back, there’s no way that I could take pills or do anything else-…I needed to know what I was dealt with, and then I had a recovery and then I got a babysitter at home just to make sure that the boys were looked after whist I was-…doing my job (Laughter) that day, you know?

Medical: I felt really, really relieved and grateful. Because of previous experiences I wanted to kind of not be in a, in a kind of hospital-y environment […]. Given the choice I didn’t want to have any sort of surgical intervention. [...] Just knowing that I had that control to do that [take the pill] myself […]. I left the clinic feeling like I’d been helped, and that was really important.

In contrast, some participants said they did not feel like they had a true choice of abortion method, often because of their pregnancy gestation. One participant said:

I thought I was one date and……I turned out to be a lot further along so it was like you’re not, you’re definitely not going to be able to have the pill……erm and then it-it kind of… so what I’d mentally prepared myself for was a pill……or erm, yeah, that sort of thing and then within a day it was, no, you’re having a surgical abortion and being put to sleep which I’d never had before.

Participants also said it was important to be offered choices around postabortion contraception. Most participants stated that they did not feel under any pressure to start contraception, and valued this. For example, a participant who preferred condoms, said the clinician “didn’t try to pressure me into anything, and none of them did, but for some reason, I felt like she really understood.”

### Accessibility

Access to abortion services influenced participant’s perceptions of care. Participants described how opportunities to reduce the time to their appointment contributed to a positive experience. Multiple participants described choosing longer travel times in order to have their abortion sooner. “Once you’ve made the decision, it’s hard to be waiting for this thing that I’m sure most women are dreading doing.” Another said that having an appointment even one day earlier made “a massive difference to my state of mind.” Several participants said they wanted an appointment sooner, and five said that the wait for their first appointment was a negative aspect of their care. For example, one participant described a long wait time until the initial phone consultation: “That was a hard six days, just waiting for this phone call. […] Especially ‘cause it’s over the phone, I feel that that could’ve been dealt with an awful lot quicker.”

In addition to the wait for their appointment, waiting times when at the clinic also impacted perceptions of care. Several participants said that the waiting times during their clinic visit were the worst part of their abortion experience, while two said the short waiting times at the clinic were the best part of their experience. One participant described the experience waiting in the clinic: “If you’re sat in a reception somewhere, erm, for a long time, I think that can possibly, like, accentuate your nerves.”

Participants who had to travel greater distances for care described how that influenced their perceptions of the abortion service. The majority who travelled said that it negatively impacted their experience. They described travelling as costly, stressful, and experienced difficulties in finding childcare or taking time off work. One participant described travelling as the worst part of her abortion experience:

Erm, it took me three and a half hours actually [...] It’s a pretty awful road as well.[...] Erm, I think that was my, the worst part of my experience actually, that I had. Actually, I don’t mind travelling to Cardiff, you know, but it’s a really long way. I’m, you know, 42, and a grown-up and I have my own car and can afford the fuel and stuff, but my thought was poor people that are in, you know- when I was a teenager, I don’t know how I would have got to Cardiff.

Several participants who had phone consultations mentioned the benefits of this modality of care, including convenience and avoiding the need to travel, arrange childcare, or make multiple visits to the clinic:

[It was] a big help to me because I’m quite far away from your clinics, and stuff, so it would’ve been a lot harder for me to get to the clinic for a consultation, and then back, and then… Do you know what I mean, backwards and forwards? It made it a lot easier being able to do it over the phone.

The contrasting experiences for participants who had to travel versus those that did not demonstrate the central role that accessibility played in positively or negatively impacting participants’ abortion experience.

## Discussion

We identified the following as key contributors to QOC amongst participants who had received abortion services in England and Wales: (1) positive interpersonal interactions, (2) being informed and prepared, (3) participating in decisions about their care, and (4) ease of access to care.

The WHO framework describes the key aspects of QOC as being safe, effective, timely, efficient, equitable and person-centred [1]. Participants of our study identified with timely, efficient, person-centred care domains of that framework. In 2015, the British Society of Abortion Care Providers (BSACP) proposed indicators of QOC in abortion for the UK [20]. Their list included: (1) percentage of abortions being performed in the first 10 weeks of pregnancy, (2) choice of procedure type (medical vs. surgical, and anaesthesia type), (3) waiting times of less than one week, (4) patient-reported measures like satisfaction surveys, and (5) engagement in quality assurance processes. While BSACP gave specific measures to their first three indicators, they didn’t propose how best to define patient-reported measures or quality assurance processes. Our results could be used to build metrics for their indicators.

Our study adds to the growing literature that moves beyond clinical conceptions of abortion QOC by exploring perceptions of abortion service quality in diverse contexts [21–26]. While specific findings of these studies differed by setting, they identified information provision and interactions with providers as critical contributors to satisfaction across contexts [21–26]. Studies also describe factors such as the comfort of facilities, privacy and confidentiality, and provider skills, as important aspects of QOC [21–26]. It is crucial that patient perspectives of abortion quality be used to inform standards and measurements.

The results of our study offer potential recommendations for improving abortion services in England and Wales and even other similar high-income country (HIC) settings. Participants consistently described how positive interactions with staff were the best part of their care, while negative interactions detracted from their experience. A recent systematic review identified over 50 studies where clinicians were assigned to participate in either an education curriculum aimed at increasing empathy and/or compassion or a control arm [27]. Authors concluded that training curricula can effectively enhance clinician empathy and compassion and recommended a framework of clinician behaviours that can improve patient perception of empathy and compassion: (1) sitting vs. standing, (2) detecting patient facial expressions and non-verbal emotional cues, (3) recognising/responding to opportunities for compassion, (4) non-verbal communication of caring, and (5) verbal statements of validation/support [27]. Clinician burnout is another contributor to poor interpersonal interactions in the healthcare setting, with some data suggesting it directly affects QOC [28, 29]. Strategies such as improved work hours and ‘wellness’ indicators can improve burnout [30]. Abortion service-providers could consider training and interventions to help their staff provide more sensitive and compassionate care given our study identified this as a critical component of QOC.

Our participants also reported that information and preparation for the abortion were important contributors to QOC. Studies in other fields of medicine have demonstrated that when information is well provided, it can improve patient satisfaction and contribute to QOC [31, 32]. Our participants described how information helped to set expectations for the abortion process, but that it was disconcerting when the expectations did not meet reality. In a secondary analysis of our study, we further explore what shapes participant preferences for a specific abortion method [33]. We identify that prior experience of abortion, accessibility, perceptions of risks, experience with a particular method, information and provider counselling are key in shaping client’s preferences. We also examine how this relates to informed consent. A qualitative study of abortion patients in Sweden described similar findings [34]. Information and preparation were closely intertwined with having choices and control over abortion options. This aligns with findings that method choice is important to abortion patients [35, 36]. Randomised clinical trials that have attempted to assign participants to medical versus surgical abortion have struggled to recruit because participants were not willing to have the choice made for them [37, 38]. Studies have also demonstrated that up to 85% have a preference for a particular abortion method before they present for care [38–40]. Service-providers should aim to maximise access to all appropriate types of abortion methods and involve them in the decision-making process to enhance QOC.

Access to abortion was another predominant contributor to QOC in our study. In particular, participants described how long wait times (both before first appointment and when at the clinic) and travel for care could contribute to negative experiences. Studies performed in settings with restrictive abortion laws and reduced access to care corroborate that these hurdles can have negative effects on patients [41–43]. A study of healthcare providers in Australia discusses ways that constrained access to abortion and other sexual and reproductive health services in rural settings can undermine the quality of care[44]. Researchers have also reported on how racial and ethnic disparities affect quality of healthcare in other high-income settings like the United States [45] and limit access to timely abortion services[46]. Our study documents perspectives on general aspects of abortion QOC in England and Wales. While it may not be feasible to remove travel and waiting times completely, service-providers can work to make access easier through modalities like telemedicine, see-and-treat pathways, and shorter targets for in-clinic wait times. In our limited sample, we were unable to explore how social factors, like income or education, and race and ethnicity, affected abortion access and quality of care. However, intersecting factors such as poverty, race, rurality, immigration status, and language limit access to—and therefore the quality of—abortion care. As such, future studies exploring quality of abortion care would benefit from adopting an intersectional approach to conceptualizing access to abortion care as a driver of quality [44, 46, 47].

Whilst our study recruited from the nation’s largest abortion provider, BPAS is part of the independent sector of abortion care and thus results may be less generalisable to other clinical settings. Another limitation of our study was its lack of ethnic diversity. All but one of our participants (86%) self-reported as white. Statistics from England and Wales report that about 77% of those having abortions in England and Wales are white [16]. While we attempted to recruit a diverse sample and selected recruitment sites that had higher populations of ethnic minority patients, we were unable to meet our target. Our inability to offer translation services to prospective participants, and thus only recruit English-speakers, further limited our aim for diversity.

## Conclusion

Across healthcare, there is a growing recognition that patient-centred outcomes are equally as important as safety and clinical outcomes [48]. It is crucial that patient perspectives of abortion QOC are used to inform standards and measurements of QOC. Further research should involve the development of patient experiences into metrics that can be measured and evaluated. Our study provides insight into the important domains of abortion QOC in England and Wales. Further research is needed to validate these findings and if they remain significant, to develop them into standardised metrics to assure quality abortion care is delivered.

## Data Availability

The datasets generated and analysed during the current study are not publicly available due to patient confidentiality.

## Abbreviations

ASQ: Abortion Service Quality
BPAS: British Pregnancy Advisory Service
BSACP: British Society of Abortion Care Providers
HIC: High-income country
LMIC: Low- and middle-income countries
NHS: National Health Service
QOC: Quality of care
WHO: World Health Organization
